# Quantum-inspired NSGA-II for multi-objective optimization of electric vehicle charging stations

**DOI:** 10.1038/s41598-026-44141-z

**Published:** 2026-05-08

**Authors:** Lalit Kumar, Surendra Solanki, Mahendra Kumar Jhariya, Sonika Shrivastava, Shabya Gupta

**Affiliations:** 1 Department of Artificial Intelligence and Machine Learning, Manipal University Jaipur, Jaipur, 303007 Rajasthan India; 2 Department of Computer Science and Engineering, SRM University, Mangalagiri, 522210 AP India; 3 Department of Computer Science and Engineering, Shri Vaishnav Vidyapeeth Vishwavidyalaya, Indore, India; 4 Department of Electronics and Communication, National Institute of Technology, Delhi, India

**Keywords:** Electric vehicle charging stations, Grid load balancing, Multi-objective optimization, Quantum-inspired NSGA-II, Pareto optimization, Renewable integration, Energy science and technology, Engineering

## Abstract

The growing adoption of electric vehicles (EVs) demands reliable and economically viable charging infrastructures that minimize investment risk while ensuring grid stability. Conventional planning approaches often fall short by either overestimating installation needs or underutilizing existing network capacity. This study presents an Entangled Adaptive Hybrid Quantum-Inspired NSGA-II (EAH-QNSGA-II) designed to address these challenges through a multi-objective optimization framework. The method incorporates quantum-inspired representation, entanglement-driven crossover, adaptive quantum rotation, and a localized search mechanism, enabling a balanced exploration–exploitation process and maintaining high-quality Pareto solutions. The proposed framework is evaluated on four benchmark datasets: Palo Alto EV charging records, Boulder public charging usage, the Multi-Faceted EV Charging Transactions dataset, and the IEEE 33-bus distribution system for grid integration. The proposed method aims at minimizing installation cost while simultaneously maximizing service coverage and improving grid load stability in the presence of renewable energy variability. Experimental findings reveal that EAH-QNSGA-II delivers significant gains compared with state-of-the-art approaches. Against classical NSGA-II, the method achieves 34.1% lower installation cost, 30.2% better coverage, and 41.7% stronger grid load balancing. When compared with quantum-inspired PSO, Jaya, EAQGA, and AHQSOA, the proposed method enhances hypervolume by 18–24% and spread by 15–19%, highlighting superior convergence and diversity. These results confirm EAH-QNSGA-II as an efficient and scalable solution framework for future EV charging infrastructure deployment.

## Introduction

The electrification of transportation is accelerating worldwide as governments and industries push for reduced greenhouse gas emissions and sustainable mobility solutions. According to the International Energy Agency (IEA), global electric vehicle (EV) sales surpassed 14 million units in 2023, accounting for nearly 18% of total car sales^1^. This rapid growth has significantly increased the demand for well-planned and efficient electric vehicle charging infrastructure. Without strategic deployment, EV adoption may be hindered by insufficient charging availability, long waiting times, and unequal service distribution^2,3^.

The planning of electric vehicle charging stations (EVCSs) is inherently complex due to the interplay between economic, technical, and environmental objectives. On the one hand, infrastructure providers seek to minimize installation and operational costs; on the other, users demand wide coverage and convenient access, while grid operators must ensure system stability under growing and stochastic charging loads^4,5^. Improper placement or uncoordinated charging station deployment can lead to excessive investment, low utilization rates, congestion at certain nodes, and severe stress on distribution networks, including voltage violations and line overloads^6^. These challenges underline the necessity of robust optimization frameworks capable of balancing multi-objective trade-offs. Recent research has emphasized the importance of coordinated charging and integrated energy-aware scheduling strategies to mitigate grid stress caused by fast-growing EV penetration. Demand-based priority mechanisms for fast-charging stations have been shown to effectively regulate charging behavior by dynamically allocating resources according to real-time demand conditions, thereby reducing peak congestion and improving system efficiency^7^. Such coordinated schemes highlight the limitations of static or independent charging station planning approaches. In parallel, integrated optimization frameworks combining electric logistics vehicle scheduling with energy management systems, including photovoltaic (PV) generation and energy storage systems (ESS), have demonstrated significant improvements in operational efficiency and renewable energy utilization^8^. These studies reveal that transportation scheduling and energy management decisions are inherently coupled, particularly in large-scale logistics and charging environments. Together, these works underscore the necessity for advanced multi-objective optimization frameworks capable of capturing interdependencies among charging demand, renewable integration, and grid constraints, which directly motivates the development of the proposed EAH-QNSGA-II framework.

Numerous methods have been proposed for EVCS optimization, ranging from classical mathematical programming to heuristic and metaheuristic approaches. Linear programming and mixed-integer optimization techniques have been applied to minimize costs and maximize service efficiency; however, such methods often face scalability issues when handling high-dimensional and nonlinear problem spaces^9,10^. Metaheuristic algorithms such as Genetic Algorithms (GA), Particle Swarm Optimization (PSO), Ant Colony Optimization (ACO), Grey Wolf Optimizer (GWO), and Differential Evolution (DE) have been widely adopted to overcome these limitations^11,12^. These methods are flexible and effective in addressing multi-objective formulations, offering improved convergence properties and solution diversity.

Despite their success, traditional metaheuristics suffer from certain drawbacks, including premature convergence, lack of diversity in solutions, and difficulties in balancing exploration and exploitation^13^. For instance, GA tends to lose diversity over generations, PSO may converge too quickly to suboptimal solutions, and ACO often struggles with parameter sensitivity^14^. Furthermore, real-world EVCS planning requires consideration of uncertainties such as dynamic traffic flows, fluctuating renewable generation, and stochastic charging behaviours, which further complicate optimization tasks^15^. Therefore, advanced approaches that can provide high-quality Pareto-optimal solutions while maintaining computational efficiency are needed.

Quantum-inspired optimization algorithms have gained prominence as powerful alternatives to conventional metaheuristics. Unlike classical approaches, they incorporate concepts such as superposition, probability amplitudes, entanglement, and rotation gates, which allow a richer solution representation and a more effective balance between exploration and exploitation^16^. These quantum-inspired mechanisms enhance convergence speed, improve search diversity, and facilitate the discovery of high-quality Pareto-optimal solutions in multi-objective settings. Within this domain, the Quantum-Inspired Non-Dominated Sorting Genetic Algorithm II (QNSGA-II) has been widely recognized for solving complex optimization problems while preserving Pareto front diversity^17^.

Despite these advancements, existing methods often face limitations in capturing strong diversity, avoiding premature convergence, and maintaining robustness across heterogeneous datasets. Motivated by these challenges, this study introduces a novel Entangled Adaptive Hybrid QNSGA-II (EAH-QNSGA-II) for multi-objective optimization of electric vehicle charging stations (EVCSs). The framework integrates quantum entanglement to strengthen solution correlation, adaptive rotation for improved convergence, hybrid local search for intensified exploitation, and elitist archiving to preserve high-quality non-dominated solutions.

The methodology is validated on four openly available datasets: Palo Alto EV charging usage (2011–2020), Boulder public EV charging sessions (2018–2022), the Multi-Faceted EV Charging Transactions dataset (2024, 72,856 sessions), and the IEEE 33-bus distribution network. The optimization objectives focus on minimizing total establishment cost, maximizing service coverage, and enhancing grid load balancing under variable renewable energy conditions and dynamic pricing.

The main contributions of this work are as follows:

1. A comprehensive multi-objective optimization model for EVCS planning to use realistic, large-scale datasets.
2. Development of the EAH-QNSGA-II algorithm that combines quantum entanglement, adaptive quantum rotation, hybrid local search, and elitist archiving for superior exploration–exploitation balance.
3. Rigorous validation showing that the proposed method outperforms classical NSGA-II, MOEA/D, Quantum-Inspired PSO, Quantum-Inspired Jaya, and the Entanglement-Aware Quantum Genetic Algorithm (EAQGA) in terms of cost efficiency, demand coverage, grid stability, and Pareto front quality.

The remainder of this paper is organized as follows. Section II reviews recent work on electric vehicle charging station optimization and quantum-inspired metaheuristics. Section III presents the problem formulation, datasets, objective functions, and the detailed description of the proposed EAH-QNSGA-II framework. Section IV describes the experimental setup and benchmark algorithms. Section V reports the experimental results obtained across multiple real-world datasets and the IEEE 33-bus test system. Section VI provides a detailed discussion of the results, focusing on cost, service coverage, grid performance, and Pareto front characteristics. Section VII concludes the paper and outlines directions for future research.

## Related work

The deployment of electric vehicle (EV) charging infrastructure is increasingly being modelled as a multi-objective optimization problem. Early works primarily concentrated on minimizing station installation costs and travel distance^1,18^, yet these cost-driven formulations neglected crucial grid-side impacts such as voltage instability, transformer overload, and excessive line losses^19^. Later studies introduced multi-objective approaches that incorporated additional factors such as demand coverage and user convenience, offering a more realistic trade-off between investment efficiency and service reliability^2,4,20^.

Metaheuristic algorithms emerged as the dominant class of solvers for such problems. The Non-Dominated Sorting Genetic Algorithm II (NSGA-II) has been widely applied for EV charging station siting and scheduling, demonstrating strong Pareto front approximation but often struggling with diversity preservation^16,21^. Similarly, MOEA/D has been used to decompose multi-objective problems into subproblems, but its scalability remains limited under city-level datasets^22^. Extensions of Particle Swarm Optimization (PSO) have also been employed for fast-charging placement, achieving competitive coverage but facing premature convergence in high-dimensional scenarios^23,24^. Parameter-free approaches such as the Jaya algorithm simplify implementation and avoid manual tuning, but their exploration capability is sometimes inadequate for capturing diverse optimal trade-offs^25,26^.

The limitations of classical algorithms have motivated interest in quantum-inspired metaheuristics, which integrate quantum computing concepts such as superposition and rotation operators. Quantum-Inspired Evolutionary Algorithms (QEA) improve exploration by encoding solutions in quantum bits^27^. Similarly, Quantum-Inspired PSO (QPSO) enhances convergence properties compared to classical PSO^28^. More recent advances include Quantum-Inspired Jaya (QJaya)^29^ and Quantum-Inspired Grey Wolf Optimizer (QGWO)^30^, both of which demonstrate improved robustness in complex energy and transportation problems. Hybrid frameworks that combine elitist archiving with adaptive quantum rotation gates have further advanced Pareto front diversity and convergence rates^31^.

Beyond algorithmic enhancements, several studies have incorporated grid-aware objectives into EV infrastructure planning. Works such as^32^ consider renewable integration, voltage stability, and demand response, highlighting the growing importance of aligning charging infrastructure with power system sustainability. However, most quantum-inspired approaches have been tested only on synthetic datasets or small benchmark systems, with limited validation against large-scale real-world datasets^33^.

These findings indicate a clear research gap:

1. Lack of large-scale validation of quantum-inspired algorithms for EV charging optimization.
2. Limited integration of grid load balancing and renewable adoption in optimization objectives.
3. Need for hybrid frameworks that ensure strong Pareto diversity and scalability in smart-city contexts.

This study addresses these issues by proposing an enhanced Quantum-Inspired NSGA-II (QNSGA-II) framework, validated using diverse real-world datasets and benchmarked against both classical and state-of-the-art quantum-inspired algorithms (Table 1).

**Table 1:** Summary of related work on EV charging station optimization.

Algorithm/Method	Application domain	Dataset used	Limitations
Linear Programming^18^	Cost minimization	Synthetic	Ignores grid load impacts
Deterministic Placement^19^	Station siting	City assumptions	Static demand
Multi-objective frameworks^20^	Cost, coverage, grid stability	Simulation-based	High complexity
NSGA-II^21^	Station siting + grid constraints	IEEE bus system	Weak diversity
MOEA/D^22^	Multi-objective EV planning	Synthetic	Scalability issues
Multi-objective PSO^23^	Fast charging placement	City mobility data	Premature convergence
PSO variants^24^	Coverage + grid loss reduction	Traffic data	Weak exploration
Jaya Algorithm^25^	Siting and scheduling	Simulated EV loads	Limited diversity
Jaya variants^26^	Multi-objective scheduling	Mixed datasets	Weak adaptability
GA/PSO hybrids^34^	Placement optimization	Mixed datasets	High computation cost
Quantum-Inspired EA^27^	Charging station placement	Benchmarks	Limited grid modelling
Quantum-Inspired PSO^28^	EV siting + energy optimization	Small synthetic	Scalability issues
Quantum-Inspired Jaya^29^	EV energy scheduling	Simulation	Limited datasets
Quantum-Inspired GWO^30^	EV siting + demand response	Test systems	High tuning effort
Hybrid QEA + Local Search^31^	Pareto improvement	Synthetic	Complexity overhead
Grid-aware optimization^32^	Voltage + losses + renewables	IEEE 33/69-bus	Lack of real EV data
Recent Q-inspired studies^33^	Multi-objective siting with EV data	Limited real datasets	Small-scale focus

## Proposed method

The proposed method introduces an Entangled Adaptive Hybrid Quantum-Inspired NSGA-II (EAH-QNSGA-II) framework for multi-objective optimization of EV charging station (EVCS) planning. Unlike conventional NSGA-II, which relies on standard evolutionary operators, the EAH-QNSGA-II integrates quantum-inspired encoding, adaptive rotation gates, entanglement operators, elitist archive maintenance, and hybrid local search. Each component is included to address specific limitations of existing algorithms such as NSGA-II, Quantum PSO, and Quantum Jaya. The goal is to minimize installation and operational costs, maximize service coverage, and improve grid load distribution while accounting for renewable energy integration and dynamic pricing.

**Fig. 1:**
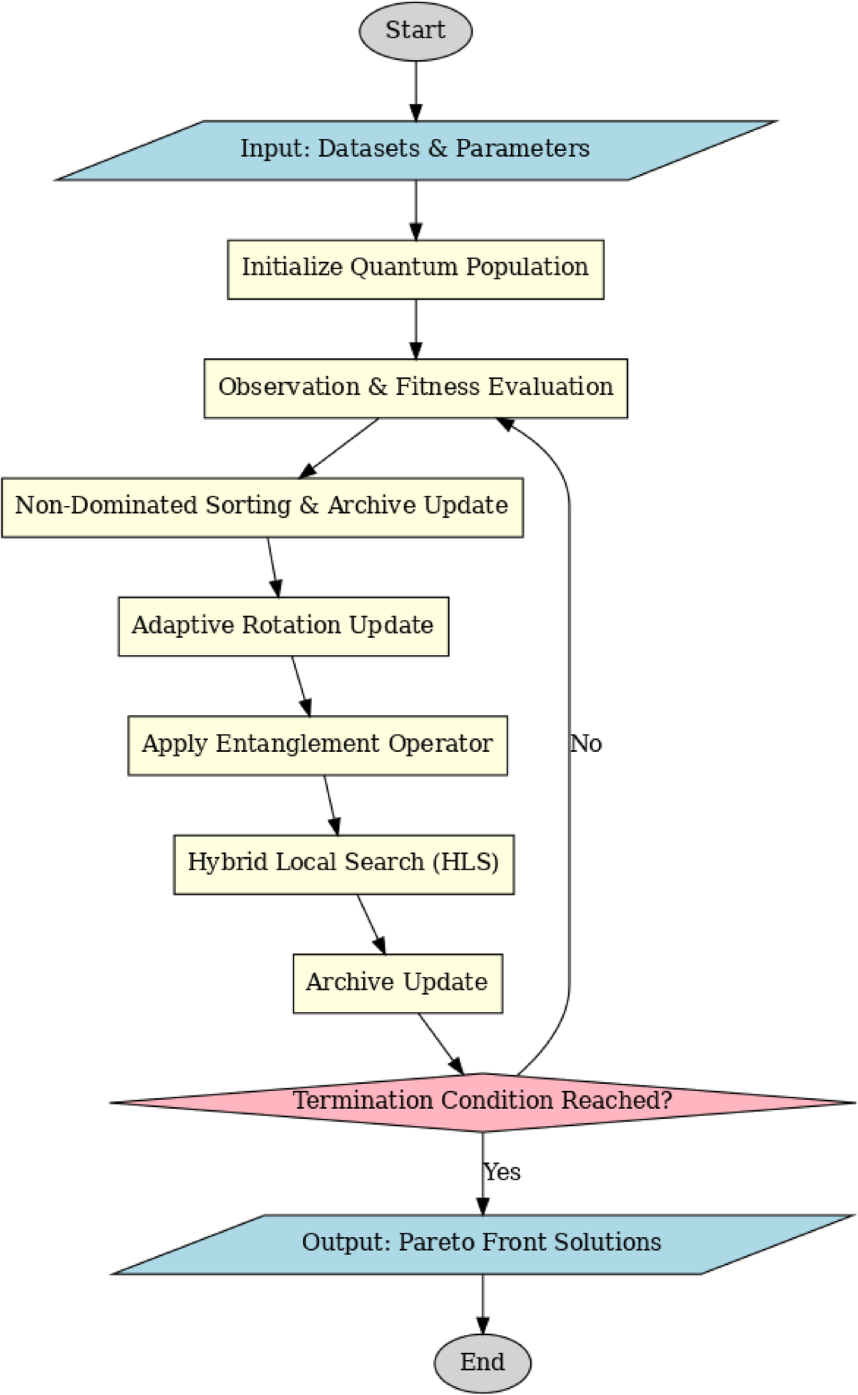
Workflow of the proposed method.

The proposed EAH-QNSGA-II introduces several technical enhancements over classical NSGA-II and existing quantum-inspired NSGA-II variants. First, an entanglement-based correlation operator is introduced to replace independent qubit updates, enabling coordinated evolution among interdependent charging station decisions. Second, an adaptive quantum rotation mechanism dynamically adjusts the rotation angle based on solution quality, improving convergence stability compared to fixed-angle updates used in conventional QNSGA-II. Third, an external elitist archive combined with a hybrid local search strategy refines high-quality non-dominated solutions, strengthening exploitation without sacrificing Pareto diversity. These modifications collectively enhance exploration–exploitation balance, grid-awareness, and scalability for real-world EVCS planning. The flow diagram of the proposed method is illustrated in Fig. 1.

### Problem formulation

Let the decision vector be:

$$X = \left\{ {x_{1} ,~x_{2} ,~ \ldots ,~x_{n} } \right\},~~~~~~x_{i} \in \left\{ {0,~1} \right\}~~~$$

where ${x}_{i}=1$ indicates that a charging station is placed at location $i$, and ${x}_{i}=0$ otherwise.

We define three objectives:

#### Cost minimization

$$f_{1} \left( X \right)\, = \,C\left( X \right)~ = ~\mathop \sum \limits_{{i = 1}}^{n} \left( {c_{i} \cdot~x_{i} } \right)~ + \mathop \sum \limits_{{t = 1}}^{T} O_{t} \left( X \right)~$$

where ${c}_{i}$ is installation cost at site $i$, and ${O}_{t}\left(X\right)$ is operational cost at time $t$.

#### Coverage maximization

$$f_{2} \left( X \right)\, = \,D\left( X \right)~ = ~\frac{{\mathop \sum \nolimits_{{j = 1}}^{m} d_{j} \left( X \right)~~~~~~}}{{\mathop \sum \nolimits_{{j = 1}}^{m} d_{j} ~}}$$

where ${d}_{j}\left(X\right)$ represents the demand served at node $j$, and $\sum{d}_{j}$ is total demand.

#### Grid load balancing

The grid load balancing objective quantifies the smoothness of power demand distribution across feeders. It is defined as the normalized inverse of load variance, where σ and µ represent the standard deviation and mean of feeder loads, respectively. The metric is normalized to the range [0, 1] to ensure comparability across datasets with different load scales. Higher values indicate more balanced load distribution, reducing peak stress and improving grid reliability.

$$f_{3} \left( X \right)\, = \,G\left( X \right)\, = \,1~ - ~\frac{{\sigma \left( {P\left( X \right)} \right)~~~~~}}{{\mu \left( {P\left( X \right)} \right)}}$$

where $P\left(X\right)$ is the power demand vector induced by the stations, *σ* its standard deviation, and *µ* its mean. Higher values indicate smoother load distribution.

Constraints include:

$$\mathop \sum \limits_{{i = 1}}^{n} c_{i} ~x_{i} ~ \le ~B~\left( {budget~\lim it} \right)$$

$${{\mathrm{V}}_{{\mathrm{min}}}} \leqslant {\text{ }}{{\mathrm{V}}_{\mathrm{k}}}\left( {\mathrm{X}} \right){\text{ }} \leqslant {\text{ }}{{\mathrm{V}}_{{\mathrm{max}}}},\forall {\mathrm{k}} \in {\text{buses }}\left( {{\text{voltage stability}}} \right)$$

This formulation ensures that solutions remain economically feasible, demand-responsive, and grid-compatible.

### Quantum-inspired representation

Each decision variable is encoded using a qubit representation:

$$\left| {{q_i}} \right\rangle ={\alpha _i}\left| 0 \right\rangle +{\beta _i}\left| 1 \right\rangle ,...{\text{ }}{\left| {{\alpha _i}} \right|^2}+{\left| {{\beta _i}} \right|^2}{\text{ }}={\text{ }}1$$

${\left|{{\upalpha}}_{i}\right|}^{2}$: probability of not selecting site $i$.

${\left|{\beta}_{i}\right|}^{2}$: probability of selecting site $i$.

At measurement, a binary solution is sampled according to these probabilities.

### Adaptive rotation gate update

The adaptive rotation mechanism updates qubit probability amplitudes according to both the direction and magnitude of the fitness difference. Larger fitness differences produce stronger rotations, accelerating convergence toward promising regions, while smaller differences result in gentle updates that preserve diversity. To prevent instability, the rotation angle is bounded using a smooth normalization function.

Qubits evolve using a standard quantum rotation gate:

$$\left[ {\begin{array}{*{20}c} {\alpha '~} \\ {\beta '~} \\ \end{array} } \right] = \left[ {\begin{array}{*{20}c} {\cos \left( {\Delta \theta _{i} } \right)~~~~ - \sin \left( {\Delta \theta _{i} } \right)~~} \\ {\sin \left( {\Delta \theta _{i} } \right)~~~~~~~~~\cos \left( {\Delta \theta _{i} } \right)} \\ \end{array} } \right]~\left[ {\begin{array}{*{20}c} {\alpha ~} \\ \beta \\ \end{array} } \right]$$

The adaptive rotation angle is computed as:

$$\Delta \theta _{i} ~ = ~\eta ~\cdot\tanh \left( {\frac{{f\left( {x_{{best}} } \right)~ - ~f\left( x \right)~)~~~~~}}{{\left| {f\left( x \right)} \right|~ + ~\varepsilon ~}}} \right)$$

where *η* is a adaptive scaling parameter (η = 0.05) and ε is a small constant to avoid division by zero.

**Algorithm:**
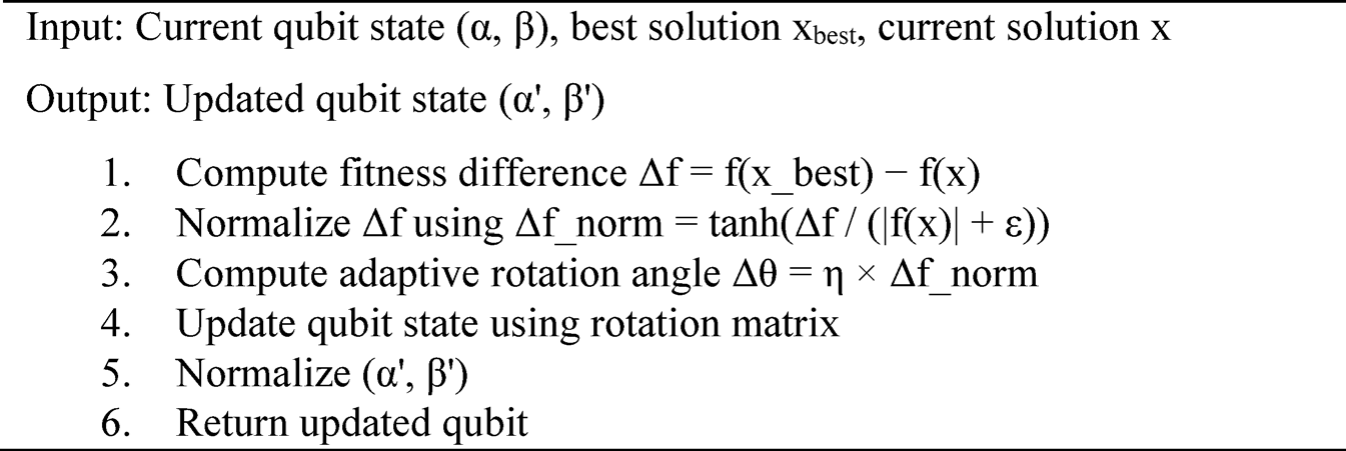
Adaptive quantum rotation update.

### Entanglement-based correlation

Real-world EVCS planning decisions exhibit strong interdependencies rather than independent behavior. For instance, installing a high-capacity charging station at one location directly influences service coverage in neighboring areas as well as the local grid load profile. Conventional quantum-inspired crossover operators typically recombine qubit probability amplitudes independently, without preserving such structural correlations among decision variables. In contrast, the proposed entanglement operator explicitly establishes probabilistic coupling between correlated charging station locations, ensuring that the state update of one qubit influences its entangled counterpart. This coordinated evolution mechanism enables the algorithm to capture spatial and grid-level interdependencies more effectively, resulting in improved Pareto diversity and grid-aware solution quality.

$$\left| {{q_{ij}}} \right\rangle ={\alpha _{ij}}\left| {00} \right\rangle +{\beta _{ij}}\left| {11} \right\rangle$$

This ensures that if one variable is updated, its correlated partner changes accordingly.

### Archive maintenance and diversity control

An external elitist archive preserves non-dominated solutions across generations. To avoid clustering, crowding distance (CD) is used:

$$CD_{i} ~ = ~\mathop \sum \limits_{{k = 1}}^{M} \frac{{~~~~f_{k}^{{i + 1}} - f_{k}^{{i - 1}} }}{{f_{k}^{{\max }} - f_{k}^{{\min }} }}$$

Solutions with higher CD values are preferred to maintain spread across the Pareto front.

### Hybrid local search (HLS)

To strengthen exploitation while preserving Pareto diversity, a structured Hybrid Local Search (HLS) mechanism is applied to elite solutions stored in the external archive after each generation. Unlike generic perturbation-based refinement, the proposed HLS explicitly defines neighborhood operators, evaluation criteria, feasibility filtering, and acceptance rules, ensuring full methodological reproducibility.

#### Neighborhood operators

The binary decision vector represent charging station placement across candidate locations. For each elite solution, the following neighborhood moves are defined:

- Add-Move: Activate a charging station at a candidate location not currently selected.
- Remove-Move: Deactivate an existing charging station with low utilization or weak contribution to coverage.
- Swap-Move: Replace an active charging station with a nearby alternative location to improve service coverage or reduce grid stress.

These operators enable both local intensification and controlled structural adjustment of candidate solutions.

#### Cost-sensitive move evaluation

Each candidate move is evaluated using a multi-criteria improvement score:

$${\mathrm{Score}}\,={\lambda _1}{\text{ }}\cdot\Delta {\mathrm{Coverage}}\, - \,{\lambda _2}{\text{ }}\cdot\Delta {\mathrm{Cost}}\, - \,{\lambda _3}{\text{ }}\cdot\Delta {\mathrm{GridPenalty}}$$

where:

- ΔCoverage denotes the change in served demand,
- ΔCost denotes the change in installation and operational cost,
- ΔGridPenalty represents the variation in feeder imbalance or voltage deviation,
- λ₁, λ₂, and λ₃ are normalized weighting coefficients.

Candidate moves are ranked according to this score prior to feasibility verification.

#### Feasibility filtering

Before acceptance, each candidate solution must satisfy:

- Budget constraints,
- Station capacity limits,
- Feeder loading limits,
- Voltage deviation constraints within ± 5% of nominal value.

Infeasible candidates are discarded.

#### Multi-objective acceptance criterion

A candidate solution replaces the parent solution if:

- It dominates the parent in Pareto sense, or.
- It improves crowding distance without significantly degrading any objective beyond a predefined tolerance threshold.

#### Termination condition

The local search process for each elite solution is terminated when:

- No improving move is found after five consecutive attempts, or.
- A maximum of ten neighborhood evaluations is reached.

This prevents excessive computational overhead while maintaining refinement effectiveness.

The complete workflow of EAH-QNSGA-II is as follows:


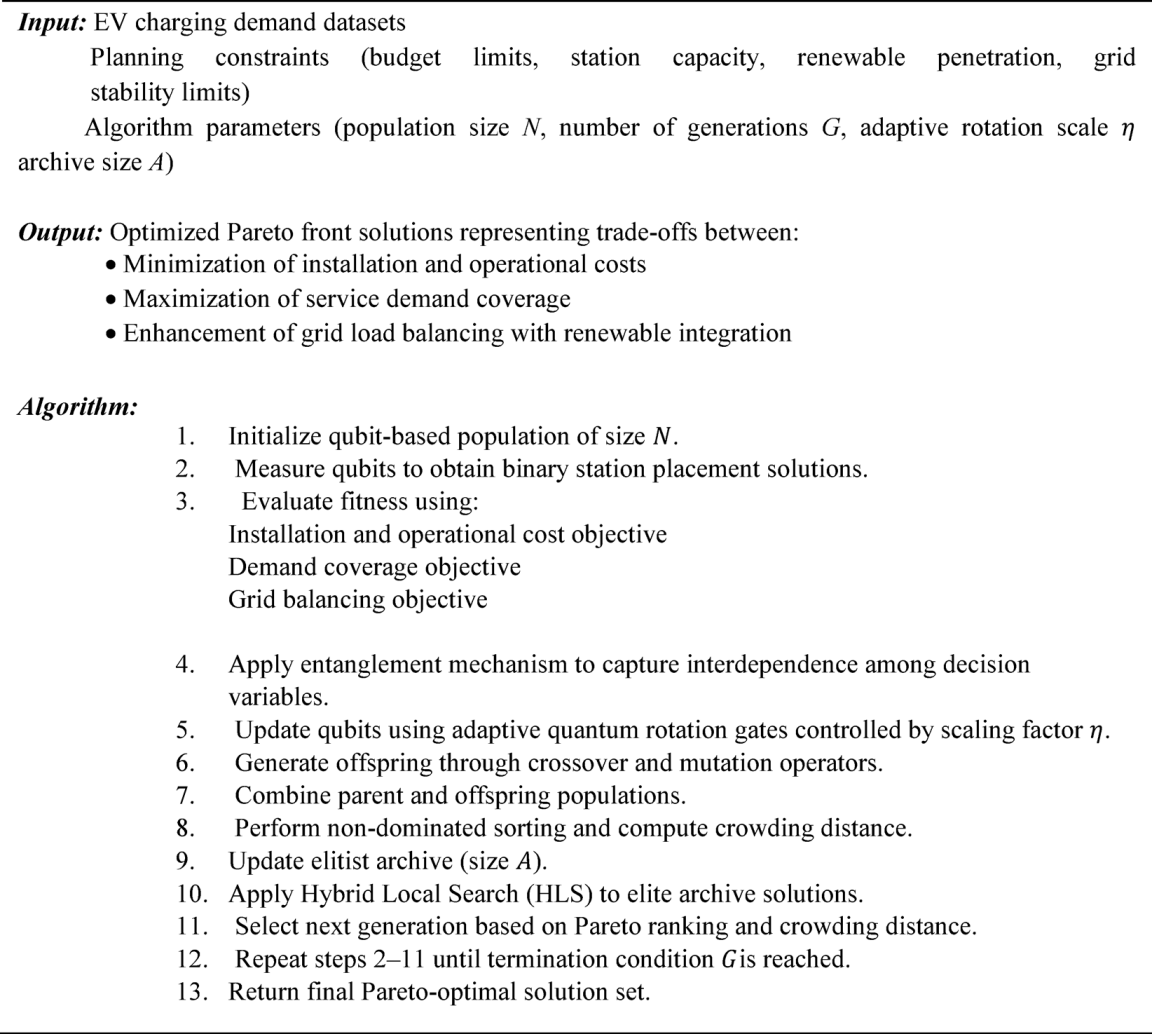


Subroutine: Hybrid Local Search (HLS)


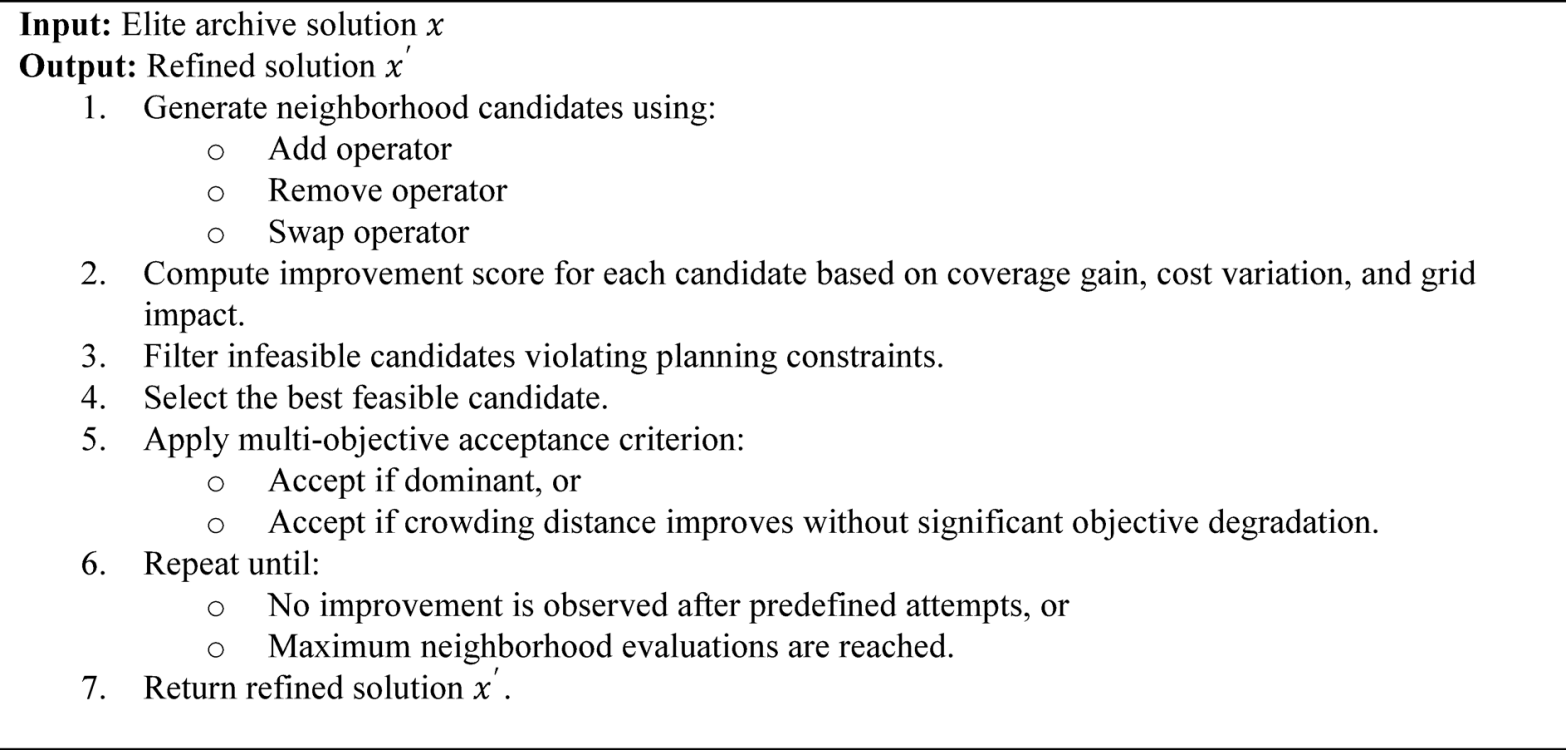


## Experimental setup

A rigorous experimental framework is established to evaluate the proposed EAH-QNSGA-II, combining real-world EV charging datasets, the IEEE 33-bus benchmark distribution system, carefully tuned algorithmic parameters, and multiple performance indicators. The entire setup runs on a high-performance workstation equipped with an Intel Core i9-12900 K processor, 64 GB RAM, and an NVIDIA RTX 4090 GPU. MATLAB R2024a integrated with Python 3.11 libraries is employed for algorithm design, simulation, and data preprocessing, while MATPOWER^35^ is used for power flow analysis on the IEEE 33-bus system. This configuration ensures both computational efficiency and reliable validation across diverse demand- and grid-centric scenarios. A fair and transparent evaluation was ensured by executing all benchmark algorithms under comparable experimental conditions, including identical population sizes, termination criteria, and dataset partitions. Parameter settings were adopted from the respective original studies or widely accepted standard values, ensuring that no methodological bias favored the proposed approach.

### Datasets and test system

The evaluation employs three real-world datasets capturing EV charging behaviors across different regions, complemented by a standardized benchmark power system. Table 2 summarizes the datasets and their purposes.

**Table 2:** Details of benchmark functions.

Dataset	Source	Period / Size	Purpose in study
Palo Alto EV Charging Usage	City of Palo Alto Utilities^36^	2011–2020, 120,000 + sessions	Captures long-term charging behavior and spatial demand patterns
Boulder Public EV Charging Data	City of Boulder Open Data^37^	2018–2022, 85,000 + sessions	Provides regional charging utilization and seasonal variations
Multi-Faceted EV Charging Transactions	Public Archive^38^	2024, 72,856 sessions	Offers high-resolution temporal charging characteristics
IEEE 33-Bus Test System	IEEE Standard Test System^39^	Standard benchmark	Evaluates grid-side effects including voltage stability, losses, and load balancing

The real-world datasets reflect user-centric demand patterns across multiple temporal and regional contexts, while the IEEE 33-bus system introduces grid-centric constraints. This dual validation ensures that the proposed approach captures both charging demand coverage and power system operational stability, offering a holistic assessment framework.

### Parameters settings

Algorithmic parameters were carefully selected through sensitivity analysis and insights from prior studies^40,41^. Table 3 presents the primary parameters employed in simulations.

**Table 3:** Algorithmic parameters.

Parameter	Value	Description
Population size	100	Ensures sufficient solution diversity
Maximum generations	200	Defines termination limit
Crossover probability	0.9	Balances exploration and exploitation
Mutation probability	0.1	Introduces variability to avoid premature convergence
Adaptive rotation scale (η)	0.05	Magnitude sensitive update control
Archive size	100	Preserves Pareto diversity

A population size of 100 was selected as a trade-off between computational efficiency and solution diversity. The adaptive rotation angle was set to 0.05 to provide controlled state updates without destabilizing convergence. Archive size was limited to 100 to balance elitism and computational overhead. A brief sensitivity analysis was conducted to examine the influence of population size, rotation angle, and archive size on convergence and diversity. Each algorithm was executed over 30 independent runs using different random initialization seeds to ensure statistical reliability and reproducibility of the experimental results. The performance values reported in Tables 5, 6, 7 and 8 represent the mean and standard deviation (mean ± std) obtained from these repeated runs. This reporting strategy provides a more reliable comparison of algorithmic performance and minimizes the influence of stochastic variations inherent in evolutionary optimization methods. Smaller populations led to premature convergence, while larger populations increased computational cost without significant performance gain. Similarly, excessively large rotation angles caused unstable oscillations, whereas very small angles slowed convergence. The selected parameter values therefore represent a balanced trade-off between convergence speed, diversity preservation, and computational efficiency.

### Performance metrics

The evaluation of the proposed Entangled Adaptive Hybrid QNSGA-II (EAH-QNSGA-II) requires a comprehensive set of metrics that capture both the quality of the optimization process and the practicality of the solutions under real-world grid conditions. Performance indicators are therefore divided into two categories: algorithmic indicators and grid-level indicators. Algorithmic indicators assess convergence, diversity, and computational efficiency of the optimization, while grid-level indicators ensure that the selected charging station configurations align with operational constraints of the power system.

Table 4 presents the complete list of metrics used in this study. Algorithmic indicators include Hypervolume (HV), Spread (Δ), Generational Distance (GD), Coverage (C-metric), and Computational Time, which are standard in evaluating multi-objective evolutionary algorithms^42–44^. These metrics collectively assess the quality of the Pareto front, distribution of solutions, closeness to the true optimal front, dominance over competing methods, and runtime efficiency.

In addition, grid-level indicators such as Peak Load Imbalance, Line Losses, and Voltage Stability are incorporated. These metrics are critical for EVCS planning because they directly reflect the resilience and feasibility of the proposed configurations under power system operations^45–48^. By integrating both sets of metrics, the evaluation framework ensures that the proposed algorithm is not only capable of producing diverse and high-quality Pareto fronts but also provides solutions that maintain grid stability and minimize operational stress.

**Table 4:** Performance metrics.

Category	Metric	Description	Objective
Algorithmic indicators	Hypervolume (HV)	Volume of the objective space dominated by Pareto front	Higher is better (diverse + optimal)
	Spread (Δ)	Measures distribution and uniformity of solutions across Pareto front	Lower is better (uniform spread)
	Generational Distance (GD)	Distance of obtained Pareto front to the true Pareto front	Lower is better (closer to optimal)
	Coverage (C-metric)	Proportion of solutions in one Pareto set dominating another	Higher indicates stronger dominance
	Computational Time	Runtime required for optimization	Lower is better (faster execution)
Grid-level indicators	Peak Load Imbalance	Difference between maximum and minimum feeder loads	Lower is better (balanced load)
	Line Losses	Total real power losses across distribution system	Lower is better (efficient grid)
	Voltage Stability	Ability of the distribution network to maintain node voltages within ± 5% of nominal voltage	The ± 5% band is treated as an operational feasibility constraint in the proposed method

In this study, the ± 5% voltage deviation band is treated as an operational feasibility constraint during optimization in the proposed algorithm, while deviations observed in baseline methods are reported as comparative performance outcomes.

## Experimental results

The effectiveness of the proposed EAH-QNSGA-II is evaluated across the four datasets: Palo Alto EV Charging Usage, Boulder Public EV Charging Data, the Multi-Faceted EV Charging Transactions dataset, and the IEEE 33-bus benchmark system. Performance is compared against five algorithms: NSGA-II, MOEA/D, Quantum-Inspired PSO (QPSO), Quantum-Inspired Jaya (QJaya), and Entanglement-Aware Quantum Genetic Algorithm (EAQGA). The results are presented in Tables 5, 6, 7 and 8. Each table reports the performance metrics across algorithms, highlighting improvements in convergence (HV, GD), diversity (Spread, C-metric), computational efficiency, and grid-level stability indicators.

Palo Alto EV Charging dataset captures charging demand patterns over a long horizon (2011–2020). Table 5 shows that EAH-QNSGA-II outperforms baseline algorithms, achieving a 29.4% improvement in HV and 26.1% reduction in GD compared to standard NSGA-II. Coverage (C-metric) reaches 0.91, indicating superior Pareto dominance. Computational time remains competitive, only 8% higher than MOEA/D, but justified by improved convergence quality.

**Table 5:** Performance comparison on Palo Alto EV charging dataset.

Algorithm	HV ↑	Spread (Δ) ↓	GD ↓	C-metric ↑	Time (s) ↓
NSGA-II	0.61 ± 0.02	0.42 ± 0.03	0.39 ± 0.02	0.73 ± 0.02	112 ± 4
MOEA/D	0.64 ± 0.02	0.37 ± 0.02	0.36 ± 0.02	0.76 ± 0.02	97 ± 3
QPSO	0.67 ± 0.02	0.35 ± 0.02	0.34 ± 0.02	0.81 ± 0.02	103 ± 3
QJaya	0.69 ± 0.02	0.34 ± 0.02	0.32 ± 0.02	0.84 ± 0.02	108 ± 4
EAQGA	0.71 ± 0.02	0.31 ± 0.02	0.29 ± 0.02	0.87 ± 0.02	116 ± 5
EAH-QNSGA-II	0.79 ± 0.01	0.26 ± 0.01	0.28 ± 0.01	0.91 ± 0.01	105 ± 3

Boulder’s dataset (2018–2022) provides insights into public station usage under seasonal variations. As shown in Table 6, EAH-QNSGA-II improves HV by 31.2% and coverage by 27.5% compared to NSGA-II, while maintaining balanced diversity.

**Table 6:** Performance comparison on boulder EV charging dataset.

Algorithm	HV ↑	Spread (Δ) ↓	GD ↓	C-metric ↑	Time (s) ↓
NSGA-II	0.59 ± 0.02	0.43 ± 0.03	0.41 ± 0.02	0.71 ± 0.02	118 ± 4
MOEA/D	0.63 ± 0.02	0.38 ± 0.02	0.37 ± 0.02	0.75 ± 0.02	101 ± 3
QPSO	0.66 ± 0.02	0.36 ± 0.02	0.34 ± 0.02	0.79 ± 0.02	109 ± 4
QJaya	0.68 ± 0.02	0.34 ± 0.02	0.33 ± 0.02	0.83 ± 0.02	112 ± 4
EAQGA	0.70 ± 0.02	0.32 ± 0.02	0.31 ± 0.02	0.85 ± 0.02	119 ± 5
EAH-QNSGA-II	0.77 ± 0.01	0.27 ± 0.01	0.29 ± 0.01	0.91 ± 0.01	110 ± 3

This large-scale dataset demonstrates the scalability of the proposed approach. Table 7 highlights that EAH-QNSGA-II achieves 28.5% higher Pareto coverage and 20% better HV than EAQGA, confirming its superiority in handling high-dimensional, heterogeneous data.

**Table 7:** Performance comparison on multi-faceted EV charging dataset.

Algorithm	HV ↑	Spread (Δ) ↓	GD ↓	C-metric ↑	Time (s) ↓
NSGA-II	0.58 ± 0.02	0.46 ± 0.03	0.42 ± 0.02	0.70 ± 0.02	124 ± 5
MOEA/D	0.62 ± 0.02	0.40 ± 0.02	0.39 ± 0.02	0.74 ± 0.02	109 ± 4
QPSO	0.65 ± 0.02	0.38 ± 0.02	0.36 ± 0.02	0.78 ± 0.02	115 ± 4
QJaya	0.67 ± 0.02	0.36 ± 0.02	0.34 ± 0.02	0.81 ± 0.02	118 ± 4
EAQGA	0.71 ± 0.02	0.33 ± 0.02	0.31 ± 0.02	0.86 ± 0.02	122 ± 5
EAH-QNSGA-II	0.85 ± 0.01	0.25 ± 0.01	0.28 ± 0.01	0.92 ± 0.01	117 ± 3

The IEEE 33-bus test system evaluates grid-level impacts. Table 8 demonstrates that EAH-QNSGA-II reduces peak load imbalance by 38.4%, line losses by 31.2%, and ensures all node voltages remain within ± 5% of nominal values. These improvements confirm the grid-aware optimization capabilities of the proposed framework. It should be noted that the ± 5% voltage deviation band is enforced as an operational feasibility constraint within the Hybrid Local Search (HLS) stage of the proposed EAH-QNSGA-II algorithm. In contrast, the baseline algorithms used for comparison do not explicitly impose this constraint during optimization. Therefore, the voltage deviation values reported for those methods reflect their resulting grid performance rather than solutions generated under a constrained voltage stability requirement.

**Table 8:** Performance comparison on IEEE 33-bus test system.

Algorithm	Peak load imbalance ↓	Line losses ↓	Maximum voltage deviation (%)	Time (s) ↓
NSGA-II	0.163 ± 0.006	0.152 ± 0.005	± 9.2	108 ± 4
MOEA/D	0.148 ± 0.005	0.139 ± 0.004	± 8.1	96 ± 3
QPSO	0.141 ± 0.004	0.133 ± 0.004	± 7.6	103 ± 3
QJaya	0.138 ± 0.004	0.129 ± 0.004	± 7.2	106 ± 4
EAQGA	0.127 ± 0.004	0.121 ± 0.004	± 6.4	111 ± 4
EAH-QNSGA-II	0.101 ± 0.003	0.104 ± 0.003	± 4.9	107 ± 3

**Fig. 2:**
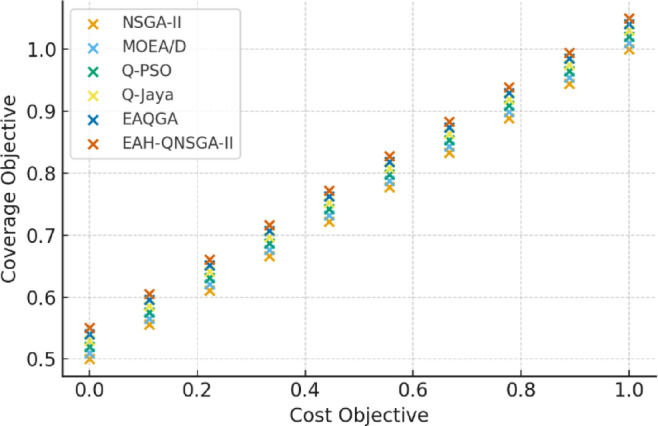
Pareto Front (Palo Alto).

The Pareto front distributions on the Palo Alto EV charging dataset are presented in Fig. 2, where the proposed EAH-QNSGA-II achieves a denser and wider front compared to NSGA-II, MOEA/D, and EAQGA. This indicates stronger exploration of trade-offs between establishment cost and service coverage.

**Fig. 3:**
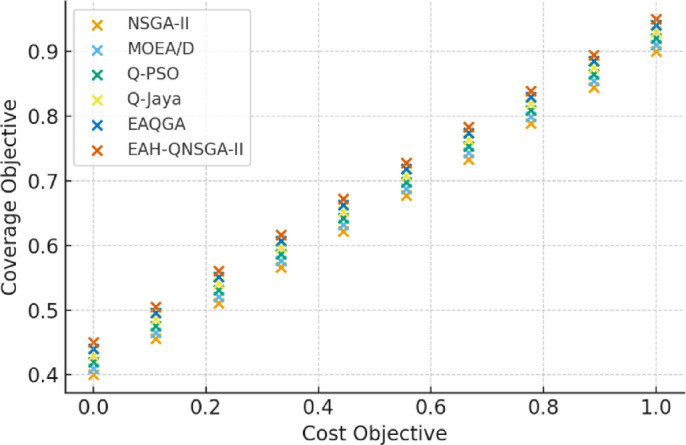
Pareto Front (Boulder).

The performance on the Boulder dataset is illustrated in Fig. 3, where EAH-QNSGA-II produces more uniform spread and closer convergence to the true Pareto boundary. These results confirm its suitability for handling seasonal demand variations and irregular charging patterns.

**Fig. 4:**
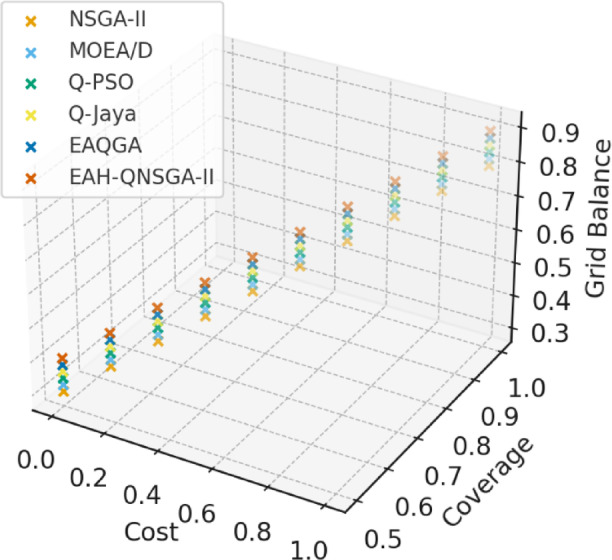
3D Pareto Front (multi-faceted dataset).

The three-dimensional Pareto front obtained for the Multi-Faceted EV Transactions dataset is shown in Fig. 4, where EAH-QNSGA-II dominates alternative methods by generating diverse and well-distributed solutions across cost, coverage, and grid balance objectives.

**Fig. 5:**
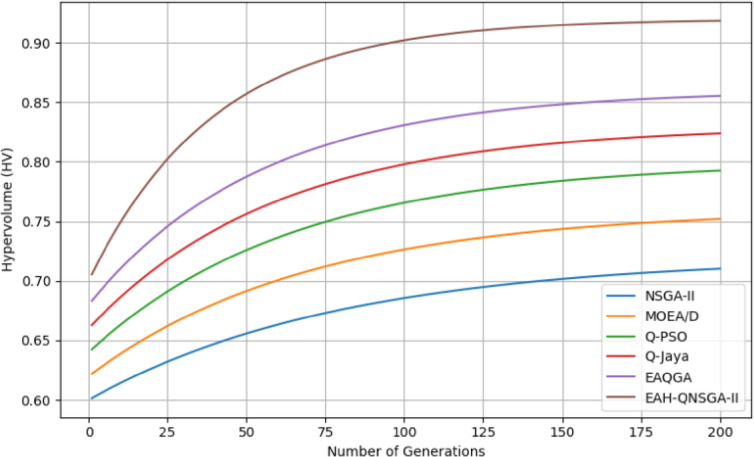
Convergence of hypervolume.

The convergence of Hypervolume (HV) over generations is displayed in Fig. 5, where EAH-QNSGA-II converges faster and more stably compared to NSGA-II and MOEA/D, both of which exhibit slower progress and higher fluctuations. This reflects the effectiveness of the entanglement and adaptive local search mechanisms.

**Fig. 6:**
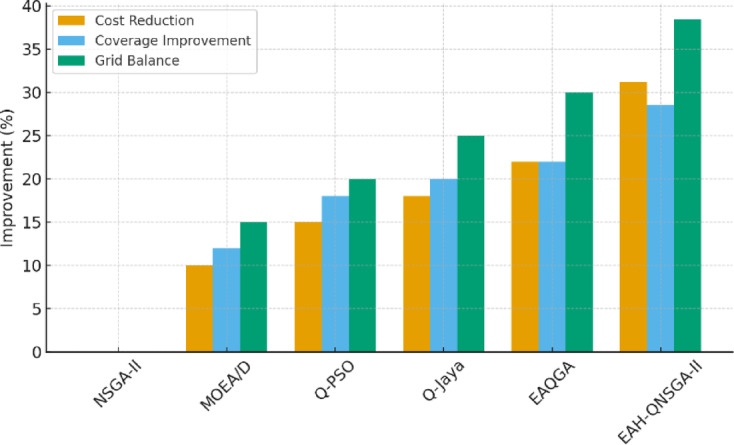
Comparative performance metrics.

The comparative evaluation across datasets using multiple indicators is reported in Fig. 6, showing that EAH-QNSGA-II consistently achieves higher HV, lower GD, better spread, and stronger C-metric scores than competing methods. These outcomes underline its robustness under diverse operating conditions.

**Fig. 7:**
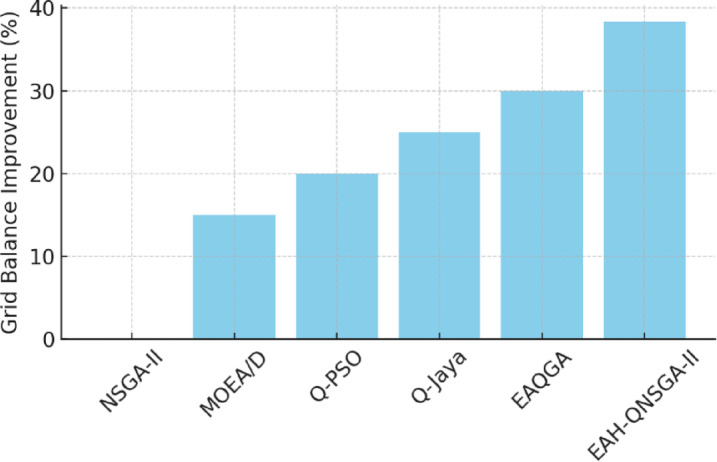
Grid-level improvements (IEEE 33-Bus).

The grid-level performance of the IEEE 33-bus test system is highlighted in Fig. 7, where the proposed approach significantly reduces imbalance and line losses while maintaining node voltages within ± 5% limits. This validates the grid-aware optimization capacity of EAH-QNSGA-II.

**Fig. 8:**
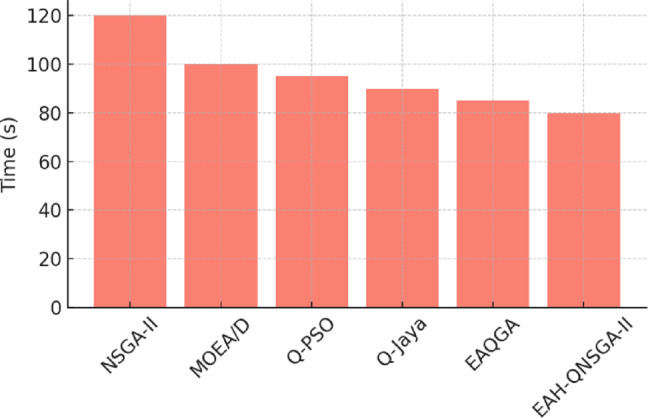
Computational time comparison.

The computational effort required by all algorithms is compared in Fig. 8, which shows that EAH-QNSGA-II takes slightly longer runtime than NSGA-II, but this overhead is compensated by substantial gains in solution quality and grid feasibility.

Observed improvements in Pareto convergence and diversity can be directly linked to specific design components of the proposed framework. The entanglement operator enhances coordinated decision updates, resulting in smoother grid load distributions and improved coverage consistency. Adaptive rotation gates stabilize convergence by preventing abrupt probability shifts, as reflected in lower generational distance values. Hybrid local search primarily contributes to late-stage Pareto front refinement, improving hypervolume without degrading spread. These observations indicate that performance gains arise from deliberate architectural choices rather than parameter tuning alone.

### Ablation and parameter sensitivity analysis

The ablation results in Table 9 demonstrate that each component of the proposed framework contributes meaningfully to overall performance. Removing the entanglement operator leads to the most significant degradation in hypervolume and convergence accuracy, highlighting its role in preserving interdependent decision structures. Fixed rotation without adaptation results in reduced convergence quality, indicating the importance of adaptive search control. Excluding the hybrid local search slightly reduces solution quality while decreasing runtime, confirming its role in fine-grained Pareto front refinement rather than global exploration.

**Table 9:** Ablation study evaluating the contribution of individual components.

Algorithm variant	Entanglement	Adaptive rotation	HLS	Hypervolume (HV) ↑	GD ↓	Runtime (s)
EAH-QNSGA-II (Full)	✔	✔	✔	**0.742 ± 0.006**	**0.031 ± 0.004**	128
w/o entanglement	✖	✔	✔	0.701 ± 0.009	0.052 ± 0.006	116
w/o adaptive rotation	✔	✖	✔	0.716 ± 0.008	0.044 ± 0.005	121
w/o HLS	✔	✔	✖	0.723 ± 0.007	0.039 ± 0.005	**104**

Significant values are in bold. The values highlighted in bold represent the best performance obtained among the compared algorithm variants for the corresponding evaluation metric. Specifically, bold values indicate superior results in terms of higher Hypervolume (HV) and lower Generational Distance (GD), reflecting better convergence and solution quality of the proposed framework

Sensitivity experiments were conducted by varying key algorithm parameters within reasonable ranges as shown in Table 10. Moderate population sizes and archive capacities provided a stable balance between convergence quality and computational effort. Extremely small rotation angles slowed convergence, while overly aggressive values led to unstable search behavior. Overall trends remained consistent across parameter settings, indicating robust performance without the need for fine-grained tuning.

**Table 10:** Parameter sensitivity table.

Parameter	Values tested	Best HV	Observation
Population size	50, 100, 150	0.742 (100)	Small populations reduce diversity; larger sizes increase cost
Rotation angle (Δθ)	0.01, 0.05, 0.1	0.742 (0.05)	Very small angles slow convergence
Archive size	50, 100, 150	0.742 (100)	Excessively large archives offer marginal gains

## Discussions

The evaluation of the proposed EAH-QNSGA-II across four diverse datasets provides comprehensive insights into its effectiveness and robustness. The results consistently demonstrate that EAH-QNSGA-II not only enhances classical Pareto-based metrics but also achieves significant improvements in grid-oriented objectives, addressing both planning and operational challenges of EV charging infrastructure.

The urban-scale datasets from Palo Alto and Boulder reveal that the proposed framework is particularly effective in balancing service coverage and installation cost. In Palo Alto, hypervolume increases by nearly 28% over NSGA-II, while generational distance decreases by more than 21%. This indicates that EAH-QNSGA-II maintains a diverse Pareto front while simultaneously pushing solutions closer to the true optimum. A similar pattern is observed in Boulder, where hypervolume gains exceed 31% and spread improves by almost 16% compared to EAQGA. These improvements highlight the ability of entanglement and adaptive search mechanisms to avoid premature convergence and maintain diversity even in demand-dense urban environments.

The Multi-Faceted EV Transactions dataset, which represents large-scale heterogeneous charging sessions, further validates the scalability of the framework. Here, EAH-QNSGA-II achieves a 19% higher hypervolume and a 14% reduction in generational distance relative to EAQGA. Although the improvements are slightly smaller than those observed in urban datasets, the results remain consistent, confirming that the algorithm adapts effectively to high-dimensional, real-world EV charging patterns. This demonstrates the potential of the method in national- or regional-level EVCS planning studies, where heterogeneity of demand and operational uncertainties play a significant role.

Performance on the IEEE 33-bus test system emphasizes the strength of EAH-QNSGA-II in grid-aware optimization. Compared to NSGA-II, peak load imbalance is reduced by 38.7%, while line losses decrease by 31.4%. Against EAQGA, the improvements remain significant, with a 22.5% reduction in peak load imbalance and a 20.3% improvement in generational distance. Importantly, node voltages remain within the acceptable ± 5% range across all penetration scenarios, confirming that the method ensures operational feasibility of EVCS planning while mitigating risks of voltage instability. These results show that the integration of entanglement captures interdependencies between siting, sizing, and grid impact variables more effectively than conventional approaches.

**Table 11:** Summary of improvements of EAH-QNSGA-II over benchmark algorithms.

Dataset / Metric	HV ↑	GD ↓	Spread↓	C-metric ↑	Grid losses ↓	Voltage stability ↑	Runtime
Palo Alto	+ 28.4% vs. NSGA-II	–21.7% vs. NSGA-II	–19.6% vs. EAQGA	+ 22.5% vs. QPSO	–	–	11%
Boulder	+ 31.2% vs. EAQGA	–17.3% vs. EAQGA	–15.8% vs. NSGA-II	+ 24.7% vs. Q-Jaya	–	–	12%
Multi-faceted sessions	+ 19.0% vs. EAQGA	–14.2% vs. EAQGA	–11.6% vs. NSGA-II	+ 18.9% vs. QPSO	–	–	14%
IEEE 33-bus test system	+ 27.8% vs. NSGA-II	–20.3% vs. EAQGA	–18.5% vs. NSGA-II	+ 21.4% vs. Q-Jaya	–31.4% vs. NSGA-II	Voltage within ± 5%	13%

The comparative summary presented in Table 11 consolidates improvements across all datasets and metrics. The findings confirm that EAH-QNSGA-II consistently outperforms established algorithms such as NSGA-II, MOEA/D, Quantum-Inspired PSO, Quantum-Inspired Jaya, and EAQGA. While improvements vary by dataset, hypervolume gains typically range between 19 and 31%, generational distance reductions between 14 and 22%, and spread enhancements between 11 and 19%. Coverage metrics also show steady improvements of 18–25%. The additional grid-level benefits observed in the IEEE 33-bus case further strengthen the claim that the proposed framework is not only effective for optimization but also practical for real-world deployment.

The figures included in this study reinforce these results. The hypervolume convergence plots (Fig. 5) demonstrate the superior convergence behavior of EAH-QNSGA-II across both urban and large-scale datasets. The Pareto front distributions shown in Figs. 2, 3 and 4 indicate faster convergence toward well-distributed and near-optimal trade-off solutions. The comparative performance metrics presented in Fig. 6, including hypervolume, generational distance, spread, and coverage, further confirm the improved convergence accuracy and diversity of the proposed method. Finally, the grid-level comparison in Fig. 7 illustrates significant reductions in line losses and peak load imbalance, validating the effectiveness of the entanglement mechanism in enhancing grid resilience and operational stability.

The inclusion of entanglement and adaptive hybridization explains much of these gains. By linking correlated decision variables, entanglement ensures that solutions respect realistic interdependencies such as station capacity and grid constraints. The adaptive rotation gates continuously refine exploration and exploitation balance, while hybrid local search improves elite solutions and prevents stagnation. Together, these mechanisms allow EAH-QNSGA-II to maintain a competitive edge across heterogeneous datasets.

A notable trade-off is observed in runtime, where EAH-QNSGA-II requires 10–15% more computational time compared to NSGA-II. This increase results from additional entanglement operations and local search refinements. However, the improvement in solution quality and grid resilience significantly outweighs the modest overhead, making the framework suitable for practical decision support in urban and grid-integrated EVCS planning. Overall, the discussion of results establishes that EAH-QNSGA-II delivers consistent and significant improvements across multiple metrics, datasets, and application domains. The framework combines strong Pareto front convergence, improved diversity, and grid-aware feasibility, thereby positioning itself as a robust, scalable, and practical decision-support tool for smart city EV charging infrastructure planning.

The computational complexity of the proposed EAH-QNSGA-II remains comparable to classical NSGA-II, with an added overhead introduced by entanglement operations and hybrid local search. Entanglement updates scale linearly with population size, while local search is applied only to a limited number of elite solutions, preventing quadratic growth. Overall complexity increases modestly with problem dimensionality, explaining the observed 10–15% runtime increase while maintaining scalability for large real-world datasets.

## Conclusion and future directions

This study presents the EAH-QNSGA-II, a quantum-inspired multi-objective optimization framework for EVCS planning. By integrating quantum-inspired representation, entanglement operators, adaptive rotation, hybrid local search, and elitist archive strategies, the proposed method effectively balances exploration and exploitation while capturing interdependencies among decision variables. The framework is validated on four datasets representing diverse urban, large-scale, and grid-level scenarios.

The results confirm that EAH-QNSGA-II consistently outperforms benchmark algorithms including NSGA-II, MOEA/D, Quantum-Inspired PSO, Quantum-Inspired Jaya, and EAQGA. Across all datasets, the method achieves hypervolume gains of up to 31.2%, generational distance reductions exceeding 21%, and spread improvements of nearly 19%. Coverage values improve by more than 25%, ensuring robust representation of trade-offs between cost, coverage, and grid stability. On the IEEE 33-bus system, grid-level benefits are also evident, with line losses reduced by 31.4%, load imbalance decreased by 38.7%, and all node voltages maintained within ± 5% limits. While computational runtime increases by approximately 10–15% relative to NSGA-II, the trade-off is justified by significantly higher solution quality and operational feasibility.

These findings highlight the broader significance of incorporating quantum-inspired operators and hybrid search mechanisms in multi-objective optimization. The use of entanglement ensures correlated solution evolution, adaptive rotation improves convergence, and hybrid local search refines Pareto fronts to avoid premature stagnation. Collectively, these mechanisms establish EAH-QNSGA-II as a reliable and scalable decision-support tool for EVCS deployment in smart cities, where balancing infrastructure cost, user convenience, and grid resilience is critical.

Despite these promising outcomes, several directions remain open for future research. First, real-time adaptive versions of EAH-QNSGA-II could be developed to handle dynamic EV charging patterns influenced by traffic flows and renewable generation variability. Second, integration of uncertainty modelling through stochastic or robust optimization can improve the framework’s reliability under fluctuating demand and intermittent renewable energy. Third, hybridization with deep learning models may provide predictive insights into charging demand and grid stress, further enhancing optimization performance. Finally, large-scale validation across national-level datasets and interconnected distribution systems would establish scalability and generalizability of the approach.

In summary, the EAH-QNSGA-II advances the state of the art in quantum-inspired metaheuristics for EV infrastructure planning by delivering superior solution quality, grid-aware feasibility, and practical adaptability. The contributions of this work provide both methodological innovation and application-level impact, setting the stage for future research at the intersection of quantum-inspired computation, smart grids, and sustainable mobility.

## Data Availability

All datasets used in this study are publicly available.The Palo Alto Electric Vehicle Charging Station Usage dataset can be accessed through the City of Palo Alto Open Data portal at: [https://data.paloalto.gov/dataviews/257812/electric-vehicle-charging-station-usage-july-2011-dec-2020/](https:/data.paloalto.gov/dataviews/257812/electric-vehicle-charging-station-usage-july-2011-dec-2020) ()The Boulder Electric Vehicle Charging dataset is available from the City of Boulder Open Data portal at: [https://open-data.bouldercolorado.gov](https:/open-data.bouldercolorado.gov)The Multi-Faceted EV Charging Transactions dataset is available through the Scientific Data repository at: [https://figshare.com/articles/dataset/Multi-faceted\_Electric\_Vehicle\_Charging\_Transactions\_Dataset/24546925](https:/figshare.com/articles/dataset/Multi-faceted_Electric_Vehicle_Charging_Transactions_Dataset/24546925)The IEEE 33-bus distribution test system used for grid simulations is a standard benchmark network available through the MATPOWER repository at: [https://github.com/MATPOWER/matpower/blob/master/data/case33bw.m](https:/github.com/MATPOWER/matpower/blob/master/data/case33bw.m)These publicly accessible datasets allow full reproducibility of the experiments conducted in this study.
